# Machine learning model for predicting urinary tract infection risk in febrile children under 3 years of age

**DOI:** 10.3389/fped.2025.1677292

**Published:** 2025-12-08

**Authors:** Le-zhen Ye, Jian-xin Sun, Jing Chen, Kuan-kuan Cen, Ye Bi, Yun-cong Lu

**Affiliations:** Department of Paediatrician, Women’s and Children’s Hospital of Ningbo University, Ningbo, China

**Keywords:** machine learning, prediction model, SHAP, urinary tract infection, UTI

## Abstract

**Objective:**

Urinary tract infection (UTI) is a common childhood infectious disease. Accurate prediction of UTI risk in febrile children enables timely intervention and helps avoid long-term complications such as renal scarring.

**Methods:**

1,556 cases of febrile children under 3 years of age were retrospectively analyzed, and feature variables were screened using LASSO regression. Seven machine learning (ML) algorithms, including Random Forest, were used to construct the UTI prediction model. The model performance was evaluated based on comprehensive indices, including area under the curve (AUC), calibration curve, and decision curve analysis, from which the optimal prediction model was selected. The SHAP method was applied to analyze the decision-making mechanism of the model.

**Results:**

Among the seven ML models, Random Forest performed best, achieving an AUC of 0.88 in the test set, an AUPRC of 0.824, optimal calibration (ICI = 0.12), and decision curve analysis showed superior performance compared to other ML algorithms. Through LASSO regression screening and SHAP analysis, seven core predictors were established: age, WBC count, previous UTI episodes, PLT, fever peak, CRP, prenatally detected renal abnormalities. These key indicators helped to construct an accurate prediction system for UTI risk in febrile children.

**Conclusions:**

The ML model constructed in this study can accurately predict UTI risk in febrile children under 3 years of age. The visual decision interpretation achieved through the SHAP framework can assist clinicians in quickly identifying high-risk children.

## 1 Introduction

Urinary tract infection (UTI) is a common childhood infectious disease (1). The clinical presentation varies significantly according to age: older children (typically aged ≥3 years) tend to present with typical symptoms of urinary tract irritation, such as urinary frequency and dysuria, which are seldom missed. In contrast, infants and young children (aged <3 years) usually lack specific manifestations and may present with fever as the primary symptom, accompanied by atypical signs, such as crying, lethargy, feeding difficulties, and growth retardation, which are frequently overlooked (2). Studies have shown that even in healthcare settings with high clinical vigilance and adequate diagnostic resources, the missed rate of febrile UTI in infants and young children remains at as high as 50%–70% (3), representing a significant diagnostic challenge. More critically, delayed treatment of UTI is associated with permanent renal scarring (4–7), particularly in febrile UTI, where the incidence of renal scarring ranges from 10% to 30% (2, 8–10). These pathological changes may lead to a significant increase in the risk of long-term complications, including hypertension and chronic kidney disease (11). Despite the clinical importance of early recognition and standardized treatment of UTI, factors such as insidious symptoms and difficulty in obtaining urine specimens in infants and children make early and accurate recognition of urinary tract infections a challenging task (12).

Previous studies have shown associations between obesity, bladder and bowel dysfunction (BBD), vesicoureteral reflux, age, vitamin D deficiency, fever (temperature ≥39°C), and UTI occurrence in children (13–18). However, these studies have been limited in two critical aspects. First, most have focused on exploring the diagnostic value of single or few clinical factors rather than comprehensive multivariable assessment. Second, many studies have relied on traditional statistical methods that typically assume linear relationships in the log-odds scale, which may not adequately capture the non-linear patterns and complex variable interactions present in clinical data without extensive manual feature engineering.

With the rapid development of artificial intelligence technology, machine learning (ML) algorithms capable of modeling non-linear relationships and complex interactions, combined with explainable AI frameworks such as SHAP, have demonstrated advantages in disease risk prediction by constructing multidimensional models while maintaining clinical transparency (19–21). In this study, we retrospectively analyzed the clinical data of 1,556 febrile children under 3 years of age and constructed a UTI risk prediction model using multiple ML algorithms. We compared the diagnostic performance of different algorithms and established an early warning system for UTI with clinical application value. This approach provides an evidence-based foundation for optimizing the diagnostic approach for fever in children.

## 2 Materials and methods

### 2.1 Data sources

A total of 4,971 febrile children admitted to the Women's and Children's Hospital of Ningbo University from January 1, 2020, to December 31, 2024, were identified by reviewing outpatient and inpatient electronic medical records. After applying predefined inclusion and exclusion criteria, 1,556 patients were included in the final analysis.

Inclusion Criteria: (1) Fever was defined as core temperature of ≥38.0°C. (2) Age 28 days to 3 years. (3) Hospitalization duration >24 h. (4) Complete clinical data.

Exclusion Criteria:

1. Cases presenting with predominant symptoms strongly suggesting alternative diagnoses, including:
  1. Respiratory diseases: persistent cough requiring antitussive therapy, tachypnea, wheezing, or respiratory distress
  2. Gastrointestinal diseases: vomiting (>3 episodes per day) or diarrhea (≥3 loose stools per day), with symptoms lasting >24 h, as the primary complaint
  3. Neurological disorders: seizures, altered consciousness, focal neurological deficits, or meningeal signs
  4. Rheumatological/autoimmune disorders: arthritis, characteristic rashes (e.g., malar rash, photosensitivity), or documented autoimmune disease history
2. Cases lacking both urinalysis and urine culture within 24 h of presentation.

This study was carried out in accordance with the Declaration of Helsinki and approved by the Medical Ethics Committee of the Women's and Children's Hospital of Ningbo University (EC2023-011).

**Figure 1:**
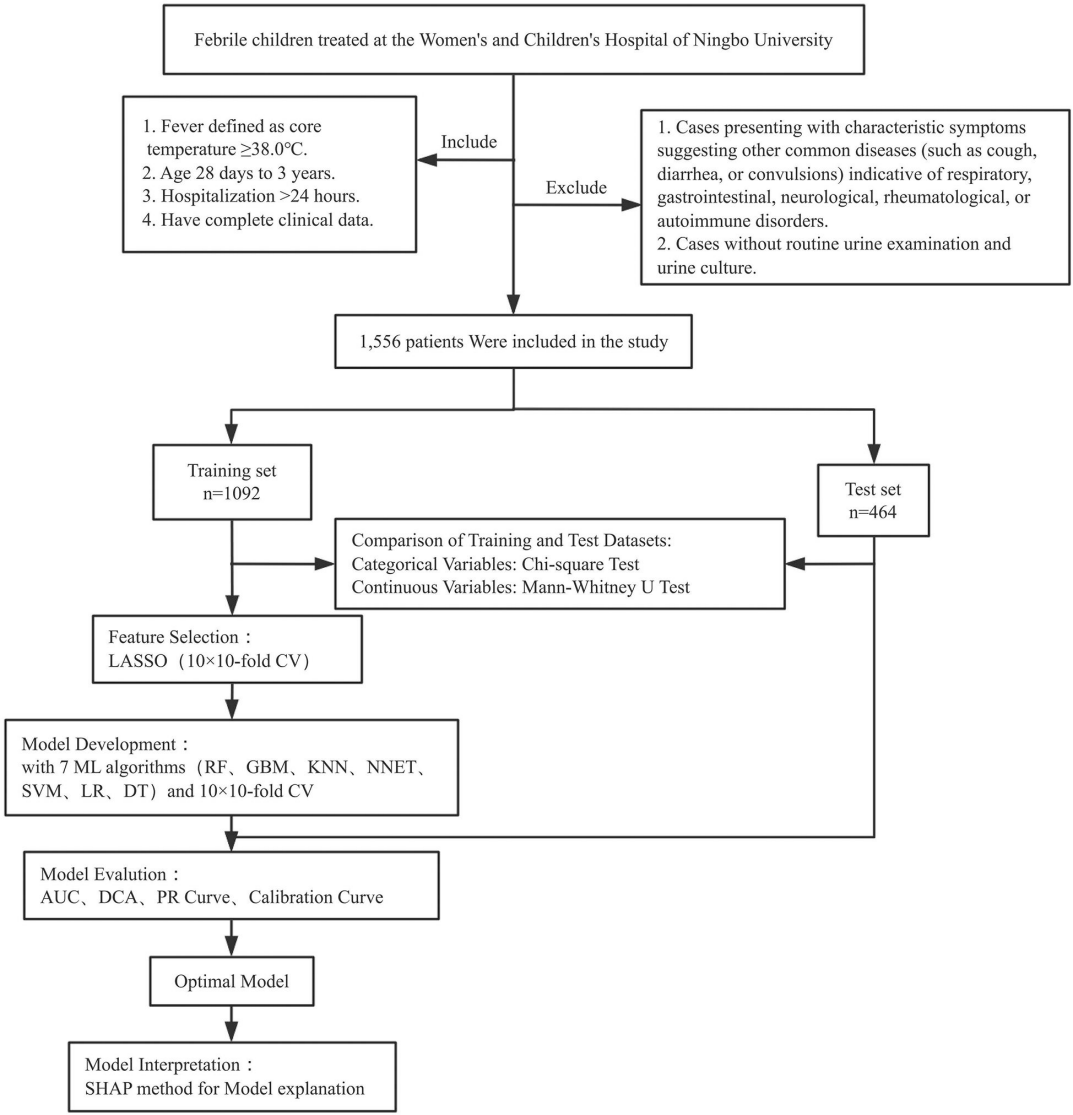
Flowchart of the methodological process for the prediction model.

Figure 1 presents the flowchart of the methodological process.

### 2.2 Methodology

This study used the R software (version 4.4.1), Python (version 3.11.9), and related extension packages for predictive model construction and evaluation.

#### 2.2.1 Study variables

Twelve variables were included: (1) Demographic and clinical characteristics, including age, sex, weight status, prenatally detected renal abnormalities, previous UTI episodes, and feeding mode. (2) Clinical symptoms, including fever peak. (3) Laboratory findings, including white blood cell count (WBC), neutrophil percentage (*N*%), hemoglobin (Hb), platelet count (PLT), C-reactive protein (CRP).

The variables were defined as follows:

1. Diagnosis of urinary tract infection: UTI was diagnosed by positive urine culture (excluding obvious contaminants) and/or ≥5 leukocytes per high-power field in centrifuged urine sediment. Positive culture was defined as ≥100,000 CFU/mL of a single pathogen for midstream specimens or ≥5 × 10^4^ CFU/mL for catheterized specimens.
2. Weight status: According to the 2006 WHO standard for evaluation of physical development of children, the interval from −1SD to +1SD was defined as normal, <−1SD as underweight, and >+1SD as overweight.
3. Prenatally detected renal abnormalities: prenatal ultrasound demonstrating abnormalities of the urinary tract including: (1) unilateral/bilateral hydronephrosis or pelvic dilatation; (2) duplicate collecting system or horseshoe kidney; (3) renal agenesis or dysplasia; (4) multicystic dysplastic kidney; and (5) ureterocele, ureteral stenosis, or ectopic ureteral insertion.
4. Fever peak: the highest value of rectal or tympanic temperature prior to admission, as reported by caregivers or documented in medical records.
5. Feeding mode: categorized by the main feeding mode within the first six months of life, including breastfeeding, mixed feeding, and artificial feeding.
6. Blood tests: Complete blood count (including WBC, *N*%, Hb, PLT) and CRP were performed at the time of presentation after the onset of fever symptoms.
7. Urinalysis and urine culture: Urine specimens were collected as early as possible after presentation, ideally before antibiotic administration. Collection methods included clean-catch midstream collection for cooperative older children, sterile urethral catheterization when clinically indicated, and bag collection for younger infants when other methods were not feasible. Urinalysis included: (1) microscopic examination for white blood cells, red blood cells, and bacteria; (2) dipstick testing for leukocyte esterase, nitrites, and protein. Urine culture referred to quantitative bacterial culture with antimicrobial susceptibility testing when indicated. When multiple urine cultures were available during the same febrile episode, the first culture result obtained within 24 h of presentation was used for analysis. Cases with discordant culture results were reviewed by two independent pediatric infectious disease specialists, with consensus determination based on clinical context, specimen quality, and colony counts.

#### 2.2.2 Sample size determination

Our sample size is rigorously justified based on both the traditional 10 EPV rule and contemporary best-practice guidelines by Riley et al. (22). Table 1 presents the calculation process, formula, and results of sample size determination.

**Table 1:** Sample size requirements for prediction model development.

Method	Sample size (*n*)	Formula
10 Events Per Candidate Predictor Parameter	365	At least 10 events per predictor parameter (EPV ≥ 10)
Four-Step Procedure		
Precise Outcome Risk Estimation	340	$n={\left(\frac{1.96}{\delta }\right)}^{2}\phi (1-\phi )$
Minimizing Prediction Error	943	$n=\exp\left(\frac{-0.508+0.259\ln(\phi )+0.504\ln(P)-\ln(\mathrm{MAPE})}{0.544}\right)$
Reducing Predictor Effect Shrinkage	549	$n=\frac{P}{(S-1)\ln\left(1-\frac{R_{cs}^{2}}{S}\right)}$
Minimizing Optimism in Model Fit	181	$\begin{array}{rl} & R_{\mathrm{Nagelkerhe}}^{2}=\frac{R_{cs}^{2}}{\max(R_{cs}^{2})},\max(R_{\mathrm{cs}}^{2})=1-\exp\left(\frac{2\ln\;L_{\mathrm{null}}}{n}\right)\\ & \ln\;L_{\mathrm{null}}=E\ln\left(\frac{E}{n}\right)+(n-E)\ln\left(1-\frac{E}{n}\right),S=\frac{R_{cs}^{2}}{R_{cs}^{2}+\delta \max(R_{cs}^{2})}\\ & n=\frac{P}{(S-1)\ln\left(1-\frac{R_{cs}^{2}}{S}\right)} \end{array}$

#### 2.2.3 Grouping methods

The caret package (version 7.0.1) was used to randomly divide all patients into training and test sets in a ratio of 7:3, where the training set contained 1,092 samples and the test set contained 464 samples. Based on the diagnostic criteria, 513 children were diagnosed with UTI (294 males, 57.3%) and 1,043 children were classified as non-UTI cases.

For comparisons between the training and test sets, continuous variables were expressed as medians and interquartile ranges and were compared using the Mann–Whitney *U* test. Categorical variables were expressed as numbers and percentages and compared using the chi-square test. Statistical significance was set at *p* < 0.05. Statistical analyses were performed using Statistical Package for the SPSS (version 29.0). Comparison of characteristics between the groups showed well-balanced baseline characteristics, with no statistically significant differences (all *p* > 0.05). Table 2 provides a comprehensive overview of the baseline characteristics between the two groups.

**Table 2:** Summary of baseline data analysis results.

Variable	Training set (*n* = 1,092)	Test set (*n* = 464)	*Z*	*p*
UTI, *n* (%)				
Yes	358 (32.8)	155 (33.4)	0.057	0.812
No	734 (67.2)	309 (66.6)		
Sex, *n* (%)				
Male	579 (53.0)	265 (57.1)	2.195	0.138
Female	513 (47.0)	199 (42.9)		
Age, *n* (%)				
<3 months	245 (22.4)	113 (24.4)	1.195	0.550
3 months to 1 year	456 (41.8)	197 (42.5)		
1–3 years	391 (35.8)	154 (33.2)		
Weight status, *n* (%)				
>+1SD	395 (36.2)	148 (31.9)	2.825	0.244
<−1SD	84 (7.7)	35 (7.5)		
−1SD to +1SD	613 (56.1)	281 (60.6)		
Fever peak, *n* (%)				
≤39°C	474 (43.4)	205 (44.2)	0.079	0.778
>39°C	618 (56.6)	259 (55.8)		
Prenatally detected renal abnormalities, *n* (%)				
Abnormal	29 (2.7)	11 (2.4)	0.106	0.745
Normal	1,063 (97.3)	453 (97.6)		
Previous UTI episodes, *n* (%)				
≥2 times	19 (1.7)	8 (1.7)	0.165	0.921
1 time	67 (6.1)	26 (5.6)		
0 time	1,006 (92.1)	430 (92.7)		
Feeding mode				
Artificial feeding	216 (19.8)	73 (15.7)	4.505	0.105
Mixed feeding	278 (25.5)	135 (29.1)		
Breastfeeding	598 (54.8)	256 (55.2)		
Anemia				
Yes	153 (14.0)	66 (14.2)	0.012	0.912
No	939 (86.0)	398 (85.8)		
WBC (×10^9^/L), median	13,600 (7,500, 18,900)	13,900 (7,833, 19,400)	−0.367	0.714
*N* (%), median	55,000 (44,000, 64,075)	56,000 (42,000, 64,000)	−0.700	0.484
PLT (×10^9^/L), median	290,000 (213,000, 378,750)	282,000 (209,250, 384,000)	−0.466	0.641
CRP (mg/L), median	27,000 (6,400, 69,500)	33,800 (7,525, 73,475)	−1.660	0.097

#### 2.2.4 Construction and evaluation of prediction models

Data screening: LASSO regression analysis was performed using the glmnet package (version 4.1.8). Through 10-fold cross-validation, the optimal *λ* value was selected based on the lambda.1se criterion, and feature variables with non-zero coefficients were screened to extract key features.

Comprehensive multi-model analysis: The caret package (version 7.0.1) was used to construct seven ML models: Logistic Regression (glm), Random Forest (rf), Gradient Boosted Tree (gbm), Neural Network (nnet), Decision Tree (rpart), Support Vector Machine (svmRadial), and K-Nearest Neighbors (knn). For model evaluation, the pROC package (version 1.18.5) was used to plot the ROC curves, the PRROC package (version 1.4) was used to analyze precision-recall curves, and decision curve analysis was used to calculate the net gain of each model under different threshold probabilities. Model calibration was evaluated using the integrated calibration index (ICI), with lower values indicating better calibration performance. These models were tested, and the performance metrics of the training and test sets were compared and analyzed to select the optimal model.

Model Interpretation: To enhance model interpretability, Python (version 3.11.9) was used to calculate the SHAP values, which were combined with feature importance ranking, swarm plots, and dependency plots to facilitate model interpretation at both global and individual levels.

## 3 Results

### 3.1 Screening of factors characterizing urinary tract infections in febrile children

**Figure 2:**
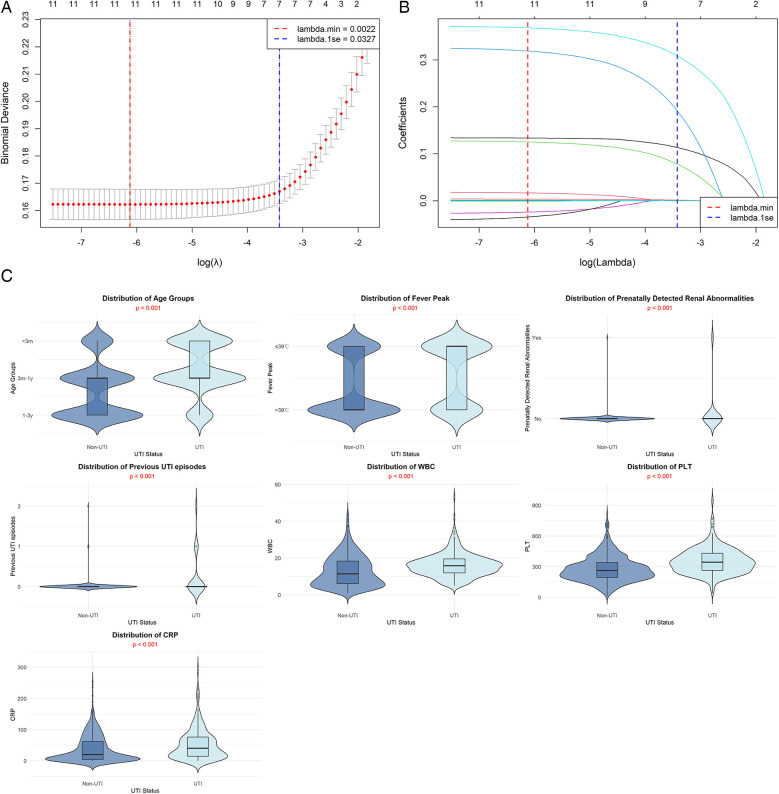
**(A)** Illustrates the variation of binomial deviance with log(*λ*), with lambda.1se chosen to balance model simplicity and prediction accuracy, ultimately retaining seven variables with non-zero coefficients. The coefficient path diagram in **(B)** shows how variable coefficients gradually shrink toward zero as log(*λ*) increases from −7 to −2 (corresponding to increasing *λ* values). This process demonstrates that: (1) high *λ* values [e.g., log(*λ*) = −2] apply strong regularization, compressing most coefficients to zero and reducing model complexity; (2) low *λ* values [e.g., log(*λ*) = −7] retain more variables but increase overfitting risk; therefore, the optimal *λ* = 0.0327 was chosen to balance prediction accuracy with clinical interpretability. **(C)** Shows the clinical features distribution comparison between UTI and non-UTI groups in the training dataset.

UTI diagnosis was the dependent variable, and 12 independent factors were subjected to the LASSO regression analysis. LASSO reduces overfitting by compressing variable coefficients through L1 regularization and addresses multicollinearity issues. Ten-fold cross-validation for the regularization parameter (*λ*) selection (Figure 2A) showed that the optimal *λ* value (lambda.1se) was 0.0327. Seven variables were ultimately selected from the 12 independent variables, including age, WBC count, previous UTI episodes, PLT, fever peak, CRP, and prenatally detected renal abnormalities. The distribution patterns of these seven LASSO-selected variables in the training dataset are shown in Figure 2C, with all features demonstrating statistically significant differences between UTI and non-UTI groups (all *p* < 0.001).

### 3.2 Analysis of machine learning models

We used seven models, namely Random Forest (RF), Gradient Boosting Tree (GBM), K-Nearest Neighbors (KNN), Neural Network (NNET), Support Vector Machine (SVM), Decision Tree (DT), and Logistic Regression (LR), for training and 10-fold cross-validation. The models were evaluated using metrics including AUC.

**Figure 3:**
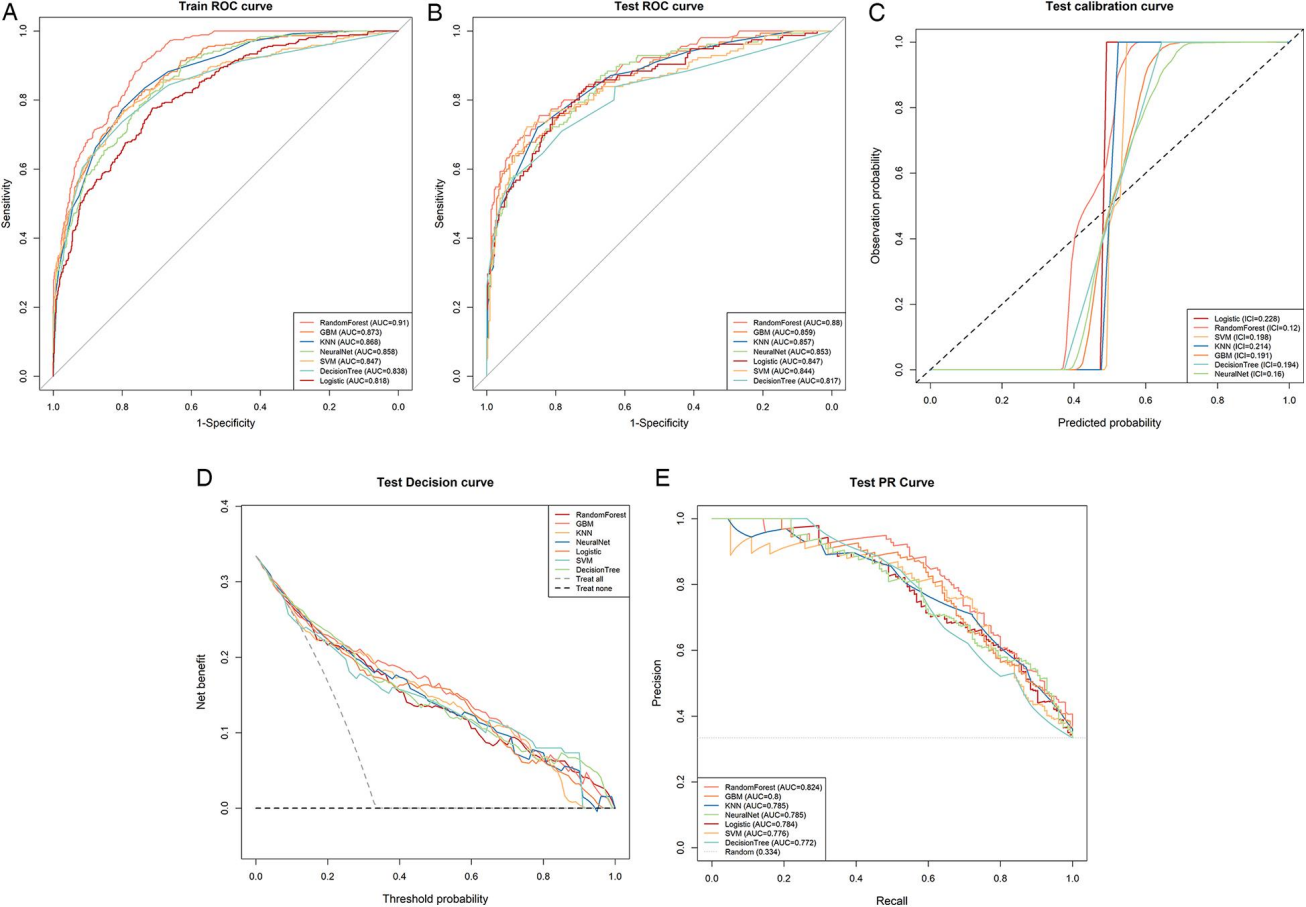
Comprehensive analysis of ML models: **(A)** training set ROC and AUC with ten-fold cross-validation. **(B)** Test set ROC and AUC. **(C)** Calibration curves for the test set: the *y*-axis represents the observed probability, the *x*-axis represents the predicted probability, and the dashed diagonal line represents the reference line. The closer the fitted line is to the reference line, the more accurate the model's predictions are. **(D)** DCA shows that RF performs better than the other ML algorithms. **(E)** Test set precision-recall (PR) curve and Average Precision (AP). The *y*-axis represents precision and the *x*-axis represents recall, and higher AP values indicate better model performance. Different colors represent different models, and the values are shown as averages.

ROC curve evaluation: In the ROC curve comparison of the training set (Figure 3A), RF (AUC = 0.91) and GBM (AUC = 0.873) achieved relatively high AUC values. In the ROC curve comparison of the test set (Figure 3B), RF (AUC = 0.88) and GBM (AUC = 0.859) performed optimally.

Calibration curve assessment: Calibration analysis revealed that all models demonstrated suboptimal calibration, with ICI values ranging from 0.120 to 0.228. Among these, the RF (ICI = 0.120) and NNET (ICI = 0.160) models showed relatively better calibration performance compared to other algorithms (Figure 3C). The calibration curves revealed systematic underestimation of UTI risk, particularly in the moderate probability range (0.4–0.6), with predicted probabilities lower than observed frequencies.

Decision curve analysis (DCA): The net benefit performance showed that the Random Forest model performed better overall. Random Forest consistently outperformed other models including Logistic Regression, KNN, SVM, and GBM in the moderate to high probability threshold range (0.2–0.8), with its curve remaining above the “Treat all” reference line in the clinically optimal range (approximately 0.1–0.6), indicating its advantages in balancing overtreatment and underdiagnosis risks.

Precision-recall curve evaluation: In the test set precision-recall curve comparison (Figure 3E), both Random Forest (AUPRC = 0.824) and GBM (AUPRC = 0.8) performed well.

The Random Forest model was identified as the optimal model based on a comprehensive evaluation of multiple metrics. Table 3 lists the detailed performance metrics of the seven models.

**Table 3:** Performance of ML models for predicting UTI.

Model	Train-AUC	Test-AUC	Test-AUPRC	Test-sensitivity	Test-specificity	Test-accuracy	ICI
RF	0.910	0.880	0.824	0.755	0.848	0.832	0.120
GBM	0.873	0.859	0.800	0.639	0.926	0.825	0.191
KNN	0.868	0.857	0.740	0.723	0.851	0.804	0.214
LR	0.818	0.847	0.784	0.748	0.809	0.802	0.228
SVM	0.847	0.844	0.776	0.723	0.883	0.812	0.198
NNET	0.858	0.853	0.785	0.716	0.828	0.810	0.160
DT	0.838	0.817	0.777	0.574	0.922	0.806	0.194

### 3.3 Interpretation of the model by SHAP

**Figure 4:**
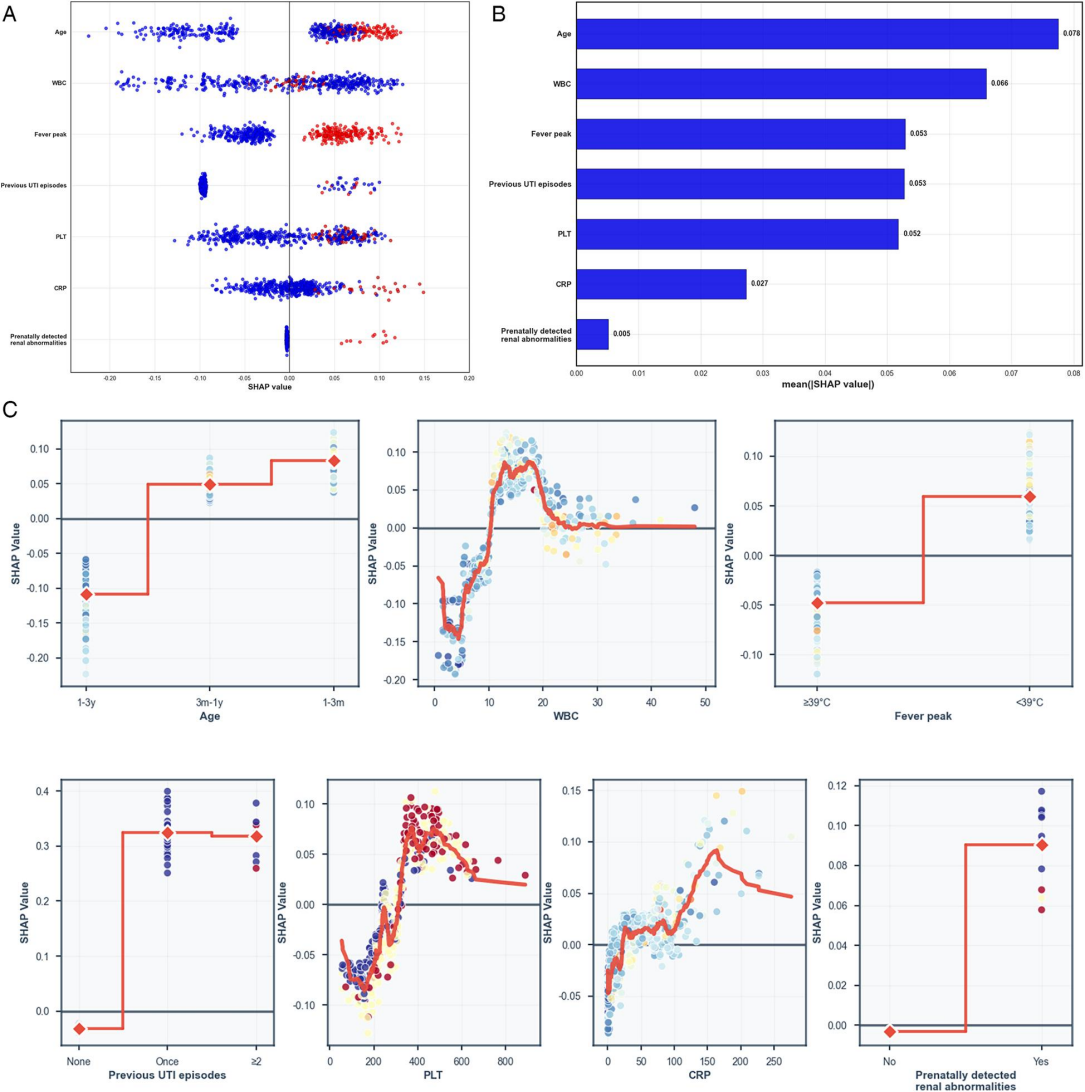
SHAP value analysis of feature importance in the random forest model. **(A)** Swarm plot showing SHAP value distribution for each feature across patient samples (red: high feature values; blue: low feature values). **(B)** Bar chart of mean absolute SHAP values ranked by feature importance. **(C)** Dependency plots demonstrating that younger age (≤1 year), elevated white blood cell count, previous UTI episodes, elevated platelet count and CRP, prenatally detected renal abnormalities, and fever peak ≤39°C were associated with increased UTI probability.

To visually explain the key predictive features in the random forest model (the final selected model based on optimal performance), we used SHAP values to quantify feature contributions, as illustrated in Figures 4A–C. In the swarm plot, each point represents the feature contribution value for a patient sample, with red points indicating high feature values and blue points indicating low feature values. The horizontal positions of the points reflect the positive or negative impact of the SHAP value on the prediction. In the bar chart, features are sorted from high to low based on their mean absolute SHAP values, and the length of the bars visually indicates the feature's importance (the longer the bar, the stronger the impact on the prediction). These two charts not only show the global ranking of feature importance, but also reveals the direction and degree of influence of each feature value on individual predictions. Figure 4C shows the dependency plots for each predictor, which demonstrate that children with younger age (especially those ≤1 year old), elevated white blood cell count, previous UTI episodes, elevated platelet count and CRP, prenatally detected renal abnormalities, and fever peak ≤39°C were more likely to be diagnosed with urinary tract infection.

### 3.4 Clinical application

Based on the above SHAP analysis results, we have developed a web-based UTI prediction system for febrile children under 3 years of age, available at https://uti-prediction.yezhiqiu.cn. By visiting this website and entering clinical indicators such as age, white blood cell count, and platelet count, users can quickly obtain UTI risk probability predictions. Furthermore, the system provides targeted medical recommendations based on the model's analysis to assist clinicians in initial screening and clinical decision-making.

## 4 Discussion

In this study, seven key predictors (age, WBC count, previous UTI episodes, PLT, fever peak, CRP, prenatally detected renal abnormalities) were screened from 12 clinical indicators by LASSO regression, and an optimal ML algorithm was identified based on multi-model comparisons for UTI prediction in febrile children. Accurate prediction of UTI risk in febrile children under 3 years of age is of great importance in clinical practice. Consequently, previous studies have examined the risk factors for urinary tract infection in children. However, no suitable ML prediction model has been developed based on these findings. A meta-analysis by Marjo Renko indicated correlations between urinary tract infections and factors including obesity, insufficient fluid intake, breastfeeding, and circumcision, but did not quantify the predictive weight of each factor (23). The ML model developed by Sriram Ramgopal's team achieved risk stratification of febrile infants and was suitable for identifying severe bacterial infections in infants up to 2 months of age but could not be used for specific prediction of urinary tract infections in children (24). Shang-Chien Li et al. constructed a UTI prediction model for febrile children under 3 years of age using traditional logistic regression analysis and developed a nomogram (25). However, this approach failed to fully exploit the advantages of ML algorithms in modeling complex feature relationships.

In this study, the SHAP method was used to provide an interpretable analysis of the ML model, demonstrating the advantages of modeling complex features. The results showed that age was an important factor in determining UTI risk. Infants and young children, especially those younger than 3 months of age, have a significantly higher incidence of UTI than children of other ages (26), which is consistent with the results of the present study.

However, our analysis revealed no significant associations between sex or weight and UTI risk, findings that contrast with established literature. Previous studies, including Tej K et al. (4), have demonstrated that females exhibit significantly higher UTI risk than males, except during early infancy. Regarding the relationship between weight status and febrile UTI, existing evidence remains controversial. While Hyung Eun Yim et al. (27) suggested that weight abnormalities (including underweight, overweight, and obesity) may increase UTI susceptibility, other studies have reported no significant association between obesity and febrile UTI risk in hospitalized young children (28). This discrepancy may be related to the fact that sex-age interactions were not analyzed in this study. Additionally, the analysis of individual indicators may have been confounded, considering that children with abnormal weight often have other comorbidities.

Elevated white blood cell counts and platelet counts are important features in predicting UTI in febrile children. Previous studies have also found that children with UTI who have elevated leukocyte and platelet counts are more likely to have renal involvement (29, 30). Leukocytosis, a common marker of inflammatory response, is associated with the risk of UTI (31). The SHAP analysis in this study not only confirmed the importance of these markers in predicting UTI occurrence but also quantified their relative contribution to the predictive model.

Previous studies have demonstrated that children with urinary tract infections under 3 months of age are more susceptible to high fever (17), which contrasts with our findings. Our study revealed that children with UTI exhibited lower peak fever temperatures compared to children with fever from other etiologies. This finding may be attributed to the earlier healthcare-seeking behavior and prompt treatment initiation in younger children, who represent the high-risk population for UTI.

In prenatal urological ultrasound examination, urinary tract dilation (UTD) is the most common abnormal finding (32). In most cases, such dilation resolves spontaneously (33); however, approximately one-third of UTD cases persist after birth or are diagnosed as congenital anomalies of the kidney and urinary tract (CAKUT) (34). In the early postnatal period, the incidence of UTI in children with UTD is estimated to be 8% to 22%. Additionally, other urinary system abnormalities, such as solitary kidney, are also considered risk factors for increased occurrence of urinary tract infections (35).

UTI should be considered in every child with fever without a source (36). Accurately identifying UTI in febrile children is a challenging clinical task, especially for younger children, whose nonspecific symptoms, insidious signs, and difficulties in urine sample collection significantly increase diagnostic difficulty (12). By developing a ML-based UTI risk prediction model, this study not only breaks through the limitations of traditional diagnostic methods but also enhances clinical utility through model interpretability analysis. Specifically, feature contribution analysis under the SHAP framework can visualize the decision weights of key predictors (e.g., age, WBC, PLT), transforming the model from a “black box” into a clinically understandable decision support tool.

This dual advantage of high-precision prediction and transparent interpretation provides clinicians with a multi-dimensional risk assessment system that helps achieve multiple clinical goals. By accurately predicting UTI risk in high-risk children, it can guide individualized antibiotic use to avoid over- or under-treatment. The system enables combining risk stratification results with the selective use of urine culture and other tests, ensuring diagnostic accuracy while reducing unnecessary testing. Additionally, clinicians can develop preventive intervention strategies based on interpretable characteristics, such as initiating early antibiotic treatment for those with elevated inflammatory markers. The integrated application of these strategies will promote pediatric UTI management from “passive treatment” to “active prevention and control”.

This study has several limitations that require attention. First, the urine collection method poses challenges, particularly for younger children under 3 months of age where bag collection is commonly used in clinical practice. While this approach is practical, it may increase the risk of sample contamination. Although catheterization significantly reduces contamination rates, its invasive nature and limited parental acceptance make routine clinical implementation difficult. Second, the retrospective single-center design presents additional constraints, as data completeness was limited by electronic medical record quality, and we did not control for potential confounders such as prior antibiotic use, which may affect the results. Multicenter prospective cohort studies would provide better validation of our findings. Third, the Random Forest model showed suboptimal calibration (ICI = 0.120) with systematic underestimation in the moderate probability range (0.4–0.6), a known limitation of tree-based ensemble methods. While this does not affect the model's discriminative ability (AUC = 0.88), absolute probability estimates should be interpreted cautiously in clinical practice. Clinicians should use model predictions as relative risk indicators to guide comprehensive assessment rather than as definitive probability estimates for individual patients.

## 5 Conclusion

This study successfully addresses a critical gap in pediatric UTI management by developing a clinically interpretable ML model for febrile children under 3 years of age. Through analysis of 1,556 cases, we identified seven key predictors using LASSO regression and demonstrated that the Random Forest model achieved superior performance (AUC = 0.88, AUPRC = 0.824, ICI = 0.12) compared to six other ML algorithms. This study introduces three principal innovations: overcoming traditional linear modeling limitations by capturing complex non-linear interactions among clinical variables; integrating the SHAP framework to transform the model from a “black box” into a transparent clinical decision support tool; and developing a web-based system that enables real-time risk assessment at the point of initial fever evaluation. These advances represent a paradigm shift from passive treatment to proactive risk-based management in pediatric UTI care, facilitating individualized antibiotic stewardship, risk-stratified diagnostic testing, and timely interventions.

While our study provides valuable insights into UTI risk assessment in febrile children younger than 3 years of age, several limitations warrant acknowledgment, including the retrospective single-center design, urine collection methodology challenges, and suboptimal calibration in moderate probability ranges. Multicenter prospective validation studies are therefore needed to refine model performance and assess the impact on clinical outcomes and cost-effectiveness. Nevertheless, this study establishes a robust foundation for ML-driven clinical decision support in pediatric infectious diseases and demonstrates the feasibility of combining accuracy with clinical interpretability to advance pediatric healthcare delivery.

## Data Availability

The raw data supporting the conclusions of this article will be made available by the authors, without undue reservation.

## References

[1] Tullus K Shaikh N. Urinary tract infections in children. Lancet. (2020) 395:1659–68. 10.1016/S0140-6736(20)30676-0 32446408 PMC13498406

[2] Brandström P Hansson S. Urinary tract infection in children. Pediatr Clin N Am. (2022) 69:1099–114. 10.1016/j.pcl.2022.07.003 36880924

[3] Bunting-Early TE Shaikh N Woo L Cooper CS Figueroa TE. The need for improved detection of urinary tract infections in young children. Front Pediatr. (2017) 5:24. 10.3389/fped.2017.00024 28271057 PMC5319447

[4] Mattoo TK Shaikh N Nelson CP. Contemporary management of urinary tract infection in children. Pediatrics. (2021) 147(2):e2020012138. 10.1542/peds.2020-012138 33479164

[5] Esposito S Biasucci G Pasini A Predieri B Vergine G Crisafi A Antibiotic resistance in paediatric febrile urinary tract infections. J Glob Antimicrob Resist. (2022) 29:499–506. 10.1016/j.jgar.2021.11.003 34801739

[6] Kane MMD. Diagnosing and treating urinary tract infections in the outpatient setting. Pediatr Ann. (2022) 51:e175–7. 10.3928/19382359-20220314-01 35575543

[7] Chandra T Bajaj M Iyer RS Chan SS Bardo DME Chen J ACR appropriateness criteria® urinary tract infection-child: 2023 update. J Am Coll Radiol. (2024) 21:S326–42. 10.1016/j.jacr.2024.02.025 38823954

[8] Shaikh N Haralam MA Kurs-Lasky M Hoberman A. Association of renal scarring with number of febrile urinary tract infections in children. JAMA Pediatr. (2019) 173:949–52. 10.1001/jamapediatrics.2019.2504 31381021 PMC6686976

[9] Gkiourtzis N Stoimeni A Glava A Chantavaridou S Michou P Cheirakis K. Prophylaxis options in children with a history of recurrent urinary tract infections: a systematic review. Pediatrics. (2024) 154:e2024066758. 10.1542/peds.2024-066758 39492618

[10] Uslu Gökceoğlu A Taş N. Renal scarring in children with febrile urinary tract infection. J Pediatr (Rio J). (2025) 101:370–4. 10.1016/j.jped.2024.10.011 39761943 PMC12039374

[11] Yang SS Tsai JD Kanematsu A Han C-H. Asian guidelines for urinary tract infection in children. J Infect Chemother. (2021) 27:1543–54. 10.1016/j.jiac.2021.07.014 34391623

[12] Marsh MC Yepes Junquera G Stonebrook E Spencer JD Watson JR. Urinary tract infections in children. Pediatr Rev. (2024) 45:260–70. 10.1542/pir.2023-006017 38689106

[13] Shaikh N Hoberman A Keren R Ivanova A Gotman N Chesney RW Predictors of antimicrobial resistance among pathogens causing urinary tract infection in children. J Pediatr. (2016) 171:116–21. 10.1016/j.jpeds.2015.12.044 26794472 PMC4808618

[14] Zaffanello M Banzato C Piacentini G. Management of constipation in preventing urinary tract infections in children: a concise review. Eur Res J. (2019) 5:236–43. 10.18621/eurj.412280

[15] Grier WR Kratimenos P Singh S Guaghan JP Koutroulis I. Obesity as a risk factor for urinary tract infection in children. Clin Pediatr (Phila). (2016) 55:952–6. 10.1177/0009922815617974 26810625

[16] Chidambaram S Pasupathy U Geminiganesan S Divya R. The association between vitamin D and urinary tract infection in children: a case-control study. Cureus. (2022) 14:e25291. 10.7759/cureus.25291 35755563 PMC9221777

[17] Lejarzegi A Fernandez-Uria A Gomez B Velasco R Benito J Mintegi S. Febrile urinary tract infection in infants less than 3 months of age. Pediatr Infect Dis J. (2023) 42:e278. 10.1097/INF.0000000000003947 37171941

[18] Balighian E Burke M. Urinary tract infections in children. Pediatr Rev. (2018) 39:3–12. 10.1542/pir.2017-0007 29292282

[19] Jordan MI Mitchell TM. Machine learning: trends, perspectives, and prospects. Science. (2015) 349:255–60. 10.1126/science.aaa8415 26185243

[20] Handelman GS Kok HK Chandra RV Razavi AH Lee MJ Asadi H. eDoctor: machine learning and the future of medicine. J Intern Med. (2018) 284:603–19. 10.1111/joim.12822 30102808

[21] Haug CJ Drazen JM. Artificial intelligence and machine learning in clinical medicine. N Engl J Med. (2023) 388:1201–8. 10.1056/NEJMra2302038 37342938

[22] Riley RD Ensor J Snell KIE Harrell FE Jr Martin GP Reitsma JB Calculating the sample size required for developing a clinical prediction model. Br Med J. (2020) 368:m441. 10.1136/bmj.m441 32188600

[23] Renko M Salo J Ekstrand M Pokka T Pieviläinen O Uhari M Meta-analysis of the risk factors for urinary tract infection in children. Pediatr Infect Dis J. (2022) 41:787–92. 10.1097/INF.0000000000003628 35788126 PMC9508987

[24] Ramgopal S Horvat CM Yanamala N Alpern ER. Machine learning to predict serious bacterial infections in young febrile infants. Pediatrics. (2020) 146:e20194096. 10.1542/peds.2019-4096 32855349 PMC7461239

[25] Li S-C Chi H Huang F-Y Chiu N-C Huang C-Y Chang L Building nomogram plots for predicting urinary tract infections in children less than three years of age. J Microbiol Immunol Infect. (2023) 56:111–9. 10.1016/j.jmii.2022.08.006 36031532

[26] Veauthier B Miller MV. Urinary tract infections in young children and infants: common questions and answers. Am Fam Physician. (2020) 102:278–85. 32866365

[27] Yim HE Han KD Kim B Yoo KH. Impact of early-life weight status on urinary tract infection in children: a nationwide population-based study in Korea. Epidemiol Health. (2021) 43:e2021005. 10.4178/epih.e2021005 33445823 PMC8060518

[28] Okada M Kijima E Yamamura H Nakatani H Yokoyama H Imai M Obesity and febrile urinary tract infection in young children. Pediatr Int. (2022) 64(1):e14686. 10.1111/ped.14686 33682248

[29] Kocaaslan R Dilli D Çitli R. Diagnostic value of the systemic immune-inflammation Index in newborns with urinary tract infection. Am J Perinatol. (2024) 41:e719–27. 10.1055/s-0042-1757353 36181758

[30] Daniel M Szymanik-Grzelak H Sierdziński J Podsiadły E Kowalewska-Młot M Pańczyk-Tomaszewska M. Epidemiology and risk factors of UTIs in children—a single-center observation. J Pers Med. (2023) 13:138. 10.3390/jpm13010138 36675799 PMC9865477

[31] Fahimi D Khedmat L Afshin A Noparast Z Jafaripor M Beigi EH Clinical manifestations, laboratory markers, and renal ultrasonographic examinations in 1-month to 12-year-old Iranian children with pyelonephritis: a six-year cross-sectional retrospective study. BMC Infect Dis. (2021) 21:189. 10.1186/s12879-021-05887-1 33602159 PMC7890627

[32] Çaltek HÖ Çaltek NC Aras D Çolak TNC Okşen E Yavuz S Prenatal diagnosis and postnatal outcomes of congenital kidney and urinary tract anomalies: results from a tertiary center. BMC Pregnancy Childbirth. (2025) 25(1):598. 10.1186/s12884-025-07723-9 40405073 PMC12096625

[33] Chiodini B Ghassemi M Khelif K Ismaili K. Clinical outcome of children with antenatally diagnosed hydronephrosis. Front Pediatr. (2019) 7:103. 10.3389/fped.2019.00103 30984723 PMC6449796

[34] Herthelius M. Antenatally detected urinary tract dilatation: long-term outcome. Pediatr Nephrol. (2023) 38(10):3221–7. 10.1007/s00467-023-05907-z 36920569 PMC10465645

[35] Herndon CDA Otero HJ Hains D Sweeney RM Lockwood GM. Perinatal urinary tract dilation: recommendations on Pre-/Postnatal imaging, prophylactic antibiotics, and follow-up: clinical report. Pediatrics. (2025) 156(1):e2025071814. 10.1542/peds.2025-071814 40518141

[36] Buettcher M Trueck J Niederer-Loher A Heininger U Agyeman P Asner S Swiss Consensus recommendations on urinary tract infections in children. Eur J Pediatr. (2021) 180(3):663–74. 10.1007/s00431-020-03714-4 32621135 PMC7886823

